# Climate warming effect of disposal fates of harvested wood products

**DOI:** 10.1186/s13021-026-00442-4

**Published:** 2026-04-21

**Authors:** Michael T. Ter-Mikaelian, Sabrina M. Desjardins, Jiaxin Chen

**Affiliations:** Ontario Ministry of Natural Resources, Ontario Forest Research Institute, 1235 Queen St. East, Sault Ste. Marie, P6A 2E5 ON Canada

## Abstract

The objective of this research was to understand how the long-term climate warming effect of harvested wood products (HWP) changed as the proportional distribution of disposed products across three primary pathways (incineration, recycling, and landfilling) varied while accounting for the possibility of multiple recycling steps. The climate warming effect is defined as greenhouse gas (GHG) emissions from the disposal of carbon content in HWP, not including emissions from processing the disposed HWP and substitution effects. Emissions were estimated over 100 years from the year of the original HWP disposal. For solid HWP, the climate warming effect decreased, for any given recycling fraction, as the landfill fraction decreased. The opposite, however, occurred for paper HWP. Recycling reduced the climate warming effect of solid HWP by “pushing” some of the emissions outside of the assessment period. For paper HWP, recycling did not change the climate warming effect unless the assessment period was relatively short. These findings can be used to assess the effect of a given combination of disposal fates of HWP, including the final disposal of non-recyclable HWP, and indicate whether it exceeds the effect of instantaneously releasing the HWP carbon content as CO_2_.

**Supplementary Information:**

The online version contains supplementary material available at 10.1186/s13021-026-00442-4.

## Introduction

Climate change is one of the most pressing challenges of our time, requiring immediate and sustained mitigation efforts across all sectors of human activity. Reducing the long-term effects of climate change has become a global priority as evidenced by the 195 signatories of the Paris Agreement [2]. According to the Sixth Assessment Report of the Intergovernmental Panel on Climate Change (IPCC), measures related to Agriculture, Forestry, and Other Land Use sector constitute some of the most substantial and cost-effective mitigation options currently available [27]. In this context, forestry-based interventions are particularly promising due to their potential for large-scale carbon (C) sequestration.

Carbon storage in harvested wood products (HWP) has emerged as a key component of forestry-based climate mitigation strategies [20, 23, 32, 33]. HWP extend the C sequestration benefits of forests beyond the ecosystem, maintaining C in solid form throughout their service life. When wood products are taken out of use, they may follow several distinct pathways, each with different climate implications [13, 32]. Some disposal options result in a relatively rapid C release, such as incineration (with or without energy recovery) or aerobic decomposition through composting. Other pathways delay emission profiles, including recycling into secondary products or deposition in landfills. In landfill environments, a substantial fraction of C in wood products may remain undecomposed for centuries, effectively creating a long-term C sink [37, 38]. Conversely, the anaerobic conditions in landfills can also generate methane (CH_4_) – a greenhouse gas with a global warming potential greater than carbon dioxide (CO_2_) – from the portion of C that does decompose.

Quantifying climate effects of wood product disposal is complicated due to the interaction between C storage, delayed release, and CH_4_ generation, and is often the subject of studies pertaining to waste management strategies. While various studies have examined the effects of HWP disposal on climate, most analyses focused on specific products, such as medium-density fiberboards and particleboards [6, 15], paper [24], or on specific disposal scenarios that reflect local or regional waste management practices (e.g., [14, 28, 31]). These product- or geographically-constrained approaches, while valuable for regional policy development, limit our understanding of how different disposal distribution patterns might affect climate benefits from wood products globally.

HWP disposal models also often assume that once the fraction of disposed HWP is recycled, the resulting product remains in use in the study system (e.g., [24, 26]). Alternatively, the product is assumed to be recycled only once, after which it can only be incinerated or landfilled (e.g., [28]). Studies that consider several recycling steps, referred to as cascading, are usually focused on the detailed analysis of a specific HWP pathway [10, 30]. Meanwhile, multiple recycling steps result in different climate warming profiles of the originally disposed product depending on the number, timing, and product fractions being recycled.

The objective of this paper was to systematically explore how the long-term climate warming effect of HWP disposal changes in response to variations in the proportional distribution of disposed products across three primary pathways (incineration, recycling, and landfilling). Here, the climate warming effect is defined as greenhouse gas (GHG) emissions from the disposal of C content in HWP, not including emissions from processing the disposed HWP and substitution effects. Instead of focusing on a given set of product disposal fractions, we evaluated the long-term climatic effect of HWP disposal across the entire space of possible combinations of the three disposal options while accounting for multiple recycling steps for the disposed product. To ensure the robustness of the results, we used (a) two different maximum number of recycling steps for solid and paper HWP and (b) two different methods (static and dynamic) of accounting for the long-term climate warming effect of methane emitted by HWP placed in the landfills. By modeling the effects on climate across multiple disposal scenarios rather than current practices alone, we aim to identify potential optimization opportunities to inform more climate-effective waste management strategies for the growing global stock of HWP.

## Methods

We estimated the climate warming effect by calculating the amount of carbon (C) released by HWP over a given time period since its disposal while considering global warming potential of C emitted as methane. We did not include emissions associated with disposal implementation, such as transporting disposed products to landfills or those from the recycling process. Similarly, we did not consider changes in emissions following substitution of other products or fuels when the HWP were recycled or incinerated for energy, or changes in forest C stock from the recycling-caused reductions in harvest volumes. These emission changes and their effect on the climate warming effect of a disposed product are discussed in the following sections.

We estimated total emissions from HWP disposal containing one unit of C in year 1. The HWP was assumed to represent solid or paper and to be “first generation”, i.e., made of virgin (not recycled) wood fiber. We assumed the lifetimes and disposal fractions were the same for virgin and recycled products regardless of how much recycling the product underwent. For recycled products, all HWP in a given category were assumed to have the same lifetime (i.e., one lifetime for all solid HWP and another one for all paper HWP).

**Fig. 1:**
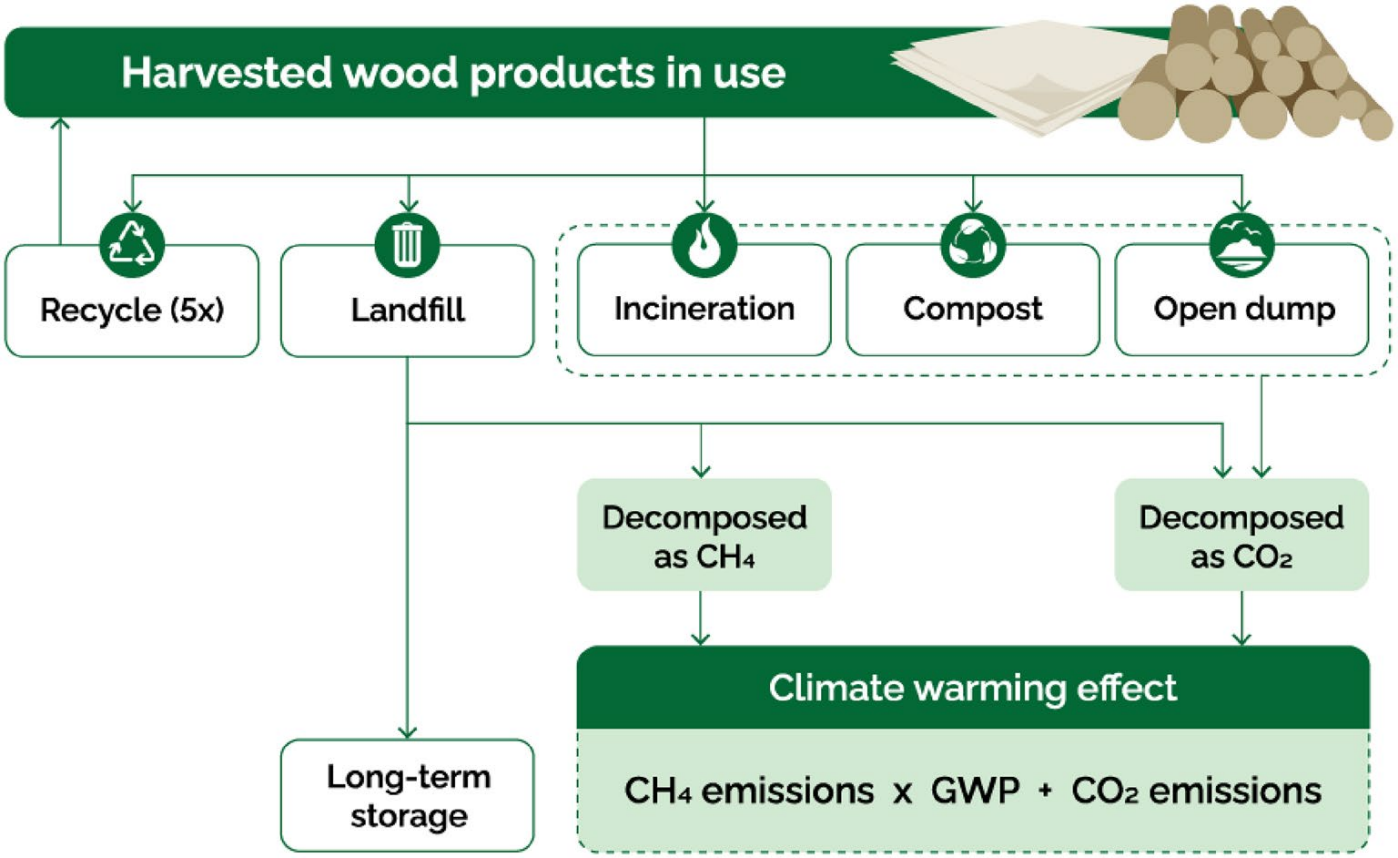
The flow of carbon from the disposed HWP

Carbon flow from the disposed HWP is depicted in Fig. 1. Disposed HWP can be incinerated (with or without energy recovery), composted, recycled, or placed in open dumps or landfills [26, 32]. Waste in shallow open dumps typically decompose aerobically with emissions containing little CH_4_ and declining faster than in anaerobic conditions [12]. Consequently, we assumed that emissions from incineration, composting, and open dump placement were strictly CO_2_. For simplicity, we also assumed that these emissions occurred in the year of disposal, from now on referring to the fraction of HWP disposed through incineration, composting, and open dump placement as burning:

1 $${B}_{0}+{W}_{0}+{R}_{0}=1$$

where *B*_*0*_, *W*_*0*_, and *R*_*0*_ are the fractions of disposed HWP allocated to burning, landfilling, and recycling, respectively.

The emissions in year *t*, *C*(*t*) (expressed in units of C), are calculated as

2 $$\begin{aligned} & C\left( t \right) = E_{CO2} \left( {0|B\left( t \right)} \right) \\& + \sum\limits_{n = 1}^{t} {\left[ {E_{CO2} \left( {t - n|W\left( n \right)} \right) + E_{CH4} \left( {t - n|W\left( n \right)} \right)} \right]} \\ \end{aligned}$$

where *B*(*t*) is the amount of HWP burned in year *t*, *W*(*n*) is the amount of HWP placed in the landfill in years *n* = 1, …, *t*, *E*_*CO2*_(0|*B*(*t*)) are CO_2_ emissions from burning HWP in year *t*, and *E*_*CO2*_(*m*|*W*(*n*)) and *E*_*CH4*_(*m*|*W*(*n*)) are landfill CO_2_ and CH_4_ emissions, respectively, *m* years after *W*(*n*) of HWP is placed in the landfill in year *n* (see Fig. S1 for the diagram showing sources of *C*(*t*)); both CO_2_ and CH_4_ emissions are presented in units of C. Recycling does not generate direct emissions but it returns the *R*_*0*_ fraction to the amount of HWP in use, some of which are disposed in year *n*, thus requiring the summation in (2).

We used IPCC Guidelines for National Greenhouse Gas inventories [12] to calculate emissions from one unit of HWP placed in a municipal landfill. Some of the HWP placed in the landfill does not decompose while the rest decomposes in either aerobic or anaerobic conditions. The HWP decomposition in anaerobic conditions generates CH_4_, some of which can be captured and subsequently combusted, either with energy generation or without it (flared). A fraction of generated CH_4_ is also oxidized into CO_2_ by methanotrophic micro-organisms when passing through soil layers or other material covering the waste [12]. The formula for CH_4_ produced in year *m*, *E*_*CH4*_(*m*), from *W*_*0*_ of HWP placed in the landfill in year 1 is as follows:

3 $$\begin{gathered} E_{CH4} \left( m \right) = W_{0} \cdot DOC_{f} \hfill \\ \cdot MCF \cdot \left( {e^{{ - \left( {m - 1} \right) \cdot k_{CH4} }} - e^{{ - m \cdot k_{CH4} }} } \right) \hfill \\ \cdot F \cdot \left( {1 - {\mathrm{Re}} c} \right) \cdot \left( {1 - OX} \right) \hfill \\ \end{gathered}$$

Here, *DOC*_*f*_ is the fraction of degradable organic C that can decompose, *MCF* is the correction factor for aerobic decomposition in the year of disposal (prior to the conditions becoming anaerobic), *k*_*CH4*_ is the CH_4_ decomposition rate, *F* is the fraction of CH_4_ in generated emissions, *Rec* is the fraction of CH_4_ recovered for energy or flaring and *OX* is the oxidation factor. The expression with exponents in brackets is for the fraction decomposed as CH_4_ in year *m* following the first order decay model commonly used to describe CH_4_ emissions from landfills [12]. The rest of the decomposed organic C is emitted as CO_2_. It should be noted that since emissions are expressed in units of C, no correction is required for the molecular weight of CH_4_.

The amount of HWP burned (*B*(*n*)) and placed in landfills (*W*(*n*)) in year *n* are calculated as fractions of *Disp*(*n*) which is the amount of HWP disposed of in year *n*; the latter comes from the HWP produced by recycling the fraction *R*_*0*_ of the original unit of HWP disposed of in year 1. In the main analysis, we assumed that both solid and paper HWP could be recycled no more than 5 times, after which they are either burned or landfilled in the same proportions as in (1) but without recycling:

4 $$\begin{gathered} B\left( n \right) = \rm B_{0} \cdot Disp\left( n \right)for \, products \, recycled \, less \, than \, 5 \, times \hfill \\ B\left( n \right) = \frac{{ B_{0} }}{{B_{0} + W_{0} }} \rm \cdot Disp\left( n \right)for \, products \, recycled \, 5 \, times \hfill \\ \end{gathered}$$

5 $$\begin{gathered} W\left( n \right) = W_{0} \rm \cdot Disp\left( n \right)for \, products \, recycled \, less \, than \, 5 \, times \hfill \\ W\left( n \right) = \frac{{ W_{0} }}{{B_{0} + W_{0} }} \cdot \rm Disp\left( n \right) \, for \, products \, recycled \, 5 \, times \hfill \\ \end{gathered}$$

Contributions to *Disp*(*n*) come from the fraction of HWP recycled in year 1 and retired *n*−1 years later; the fraction of HWP recycled twice (first time in year 1, retired out of use *l*_*1*_ years later, *R*_*0*_ of which was recycled again and then retired out of use *l*_*2*_ years later, with *l*_*1*_ + *l*_*2*_ = *n*−1); and so on. Thus, for a given number of recycles *i*, the fraction of the original product disposed of in year *n* equals the product of *R*_*0*_ to the power *i* and the fractions of the product retired after *l*_*1*_, …, *l*_*i*_ years for all possible combinations for which *l*_*1*_ + … + *l*_*i*_ = *n*−1; here, *l*_*1*_, …, *l*_*i-1*_ correspond to the periods between recycles while *l*_*i*_ is the number of years after the last recycle. For example, for *n*−1 = 3, possible sequences of events leading to the amount of product recycled in year 4 are: *R*_*0*_–3, *R*_*0*_–2–*R*_*0*_–1, *R*_*0*_–1–*R*_*0*_–2, and *R*_*0*_–1–*R*_*0*_–1–*R*_*0*_–1. Here, *R*_*0*_ indicates the act of recycling while the digits correspond to the number of years between recycling or the number of years in use after the last recycling. It is assumed that recycling (and burning and landfilling) occurs in the same year as product retirement. The general formula for the fraction of the original unit of HWP landfilled in year *n* is:

6 $$\begin{aligned} & W\left( n \right) = {Ret}\left( {n - 1} \right) \cdot R_{0} \cdot W_{0} +\\& \sum\limits_{{l_{1} = 1}}^{{\left( {n - 1} \right) - 1}} Ret\left( {l_{1} } \right) \cdot {Ret} \left( {n - 1 - l_{1} } \right) \cdot R_{0}^{2} \cdot W_{0} + \\& \sum\limits_{{l_{1} = 1}}^{{\left( {n - 1} \right) - 2}} {\sum\limits_{{l_{2} = 1}}^{{\left( {n - 1} \right) - 1 - l_{1} }} Ret } \left( {l_{1} } \right) \cdot {Ret} \left( {l_{2} } \right) \cdot \\& {Ret} \left( {n - 1 - l_{1} - l_{2} } \right) \cdot R_{0}^{3} \cdot W_{0} + \\& \sum\limits_{{l_{1} = 1}}^{{\left( {n - 1} \right) - 3}} {\sum\limits_{{l_{2} = 1}}^{{\left( {n - 1} \right) - 2 - l_{1} }} {\sum\limits_{{l_{3} = 1}}^{{\left( {n - 1} \right) - 1 - l_{1} - l_{2} }} Ret } } \left( {l_{1} } \right) \cdot \\& {Ret} \left( {l_{2} } \right) \cdot {Ret} \left( {l_{3} } \right) \cdot {Ret} \left( {n - 1 - l_{1} - l_{2} - l_{3} } \right) \cdot R_{0}^{4} \cdot W_{0} \\ & \sum\limits_{{l_{1} = 1}}^{{\left( {n - 1} \right) - 4}} {\sum\limits_{{l_{2} = 1}}^{{\left( {n - 1} \right) - 3 - l_{1} }} {\sum\limits_{l = 1}^{{\left( {n - 1} \right) - 2 - l_{1} - l_{2} }} {\sum\limits_{{l_{4} = 1}}^{{\left( {n - 1} \right) - 1 - l_{1} - l_{2} - l_{3} }} Ret}}} (l_{1} ) \cdot \\ & {Ret}(l_{2} ) \cdot {Ret} (l_{3} ) \cdot {Ret} (l_{4} ) \cdot \\& {Ret}(n - 1 - l_{1} - l_{2} - l_{3} - l_{4} ) \cdot \\ & R_{0}^{5} \cdot W_{0} /(B_{0} + W_{0} ) \end{aligned}$$

where *Ret*(*l*) is the fraction of HWP retired *l* years after its production. HWP retirement is commonly described with a negative exponential function [12]. If the retirement rate is equal to *k*_*ret*_, then

7 $${\mathrm{Re}} t\left( l \right) = e^{{ - \left( {l - 1} \right) \cdot k_{ret} }} - e^{{ - l \cdot k_{ret} }} = \left( {1 - S} \right) \cdot S^{l - 1}$$

where denotation *S* = exp(—*k*_*ret*_) is used for simplicity. Substituting (7) in (6) results in:

8 $$\begin{aligned} & W\left( n \right) = S^{n - 2} \cdot \left( {1 - S} \right) \cdot R_{0} \cdot W_{0} \\ & + \left( {_{1}^{n - 2} } \right) \cdot S^{n - 3} \cdot \left( {1 - S} \right)^{2} \cdot R_{0}^{2} \cdot W_{0} \\ & + \left( {_{2}^{n - 2} } \right) \cdot S^{n - 4} \left( {1 - S} \right)^{3} \cdot R_{0}^{3} \cdot W_{0} \\ & + \left( {_{3}^{n - 2} } \right) \cdot S^{n - 5} \left( {1 - S} \right)^{4} \cdot R_{0}^{4} \cdot W_{0} \\ &+ \left( {_{4}^{n - 2} } \right) \cdot S^{n - 6} \left( {1 - S} \right)^{5} \cdot R_{0}^{5} W_{0} /\left( {B_{0} + W_{0} } \right) \end{aligned}$$

where *n* over *k* in brackets denotes the number of combinations of *k* objects from a set with *n* objects. Formula (8) applies to *n* > 5; for *n* = 2, …., 5, it simplifies to

9 $$W\left(n\right)=(1-S)\cdot {((1-S)\cdot {R}_{0}+S)}^{n-2}\cdot {R}_{0}\cdot {W}_{0}$$

For *n* = 1 the fraction of HWP placed in the landfill is equal to *W*(1) = *W*_*0*_ as defined above. For the formulae describing the fraction of HWP burned in year *n*, *B*(*n*), all instances of *W*_*0*_ are replaced with *B*_*0*_ in (8) and (9) except for the denominator in the last term in (8).

Formulae (6–9) reflect the assumption of maximum 5 recycling steps for both solid and paper HWP. We also considered the alternative maximum number of recycling steps to reflect scenarios commonly analyzed in the literature on product disposal, namely: 2 and 7 recycling steps for solid and paper HWP, respectively. The formulae derivation and results for the alternative number of recycling steps are in Supplementary Information.

Formula (2) provides the amount of C emitted in year t as CO_2_ and CH_4_. A common approach to account for the warming effects of CH_4_ is to multiply the global warming potential of CH_4_ calculated over 100 years, *GWP*_*100*_. The sum of Eq. (2) with the term for CH_4_ multiplied by *GWP*_*100*_ provides an estimate of the total emission effect over the period *T* for one unit of C in virgin HWP disposed of in year 1:

10 $$\begin{aligned}& C_{Eq\_total} \left( T \right) = \sum\limits_{t = 1}^{T} {E_{CO2} \left( {0|B\left( t \right)} \right)} \\& + \sum\limits_{t = 1}^{T} {\sum\limits_{n = 1}^{t} {\left[ {E_{CO2} \left( {t - n|W\left( n \right)} \right) + GWP_{100} \cdot E_{CH4} \left( {t - n|W\left( n \right)} \right)} \right]} }\end{aligned}$$

Here, *C*_*Eq_total*_(*T*) is the equivalent of CO_2_eq commonly used to describe the climate warming effect of various GHGs. Note that it is transformed back to the original C mass units to make the effect easily assessable; if *C*_*Eq_total*_(*T*) is less than 1 then the warming effect of the disposed HWP is less than if its entire C content was emitted as CO_2_, and vice versa.

The formula for *C*_*Eq_total*_(*T*) applies to solid and paper HWP, but the parameter values are different. In the analysis, we used the default values from IPCC [12]. For solid HWP, parameters were the retirement rate *k*_*ret*_ = 0.0198 corresponding to 35 years of product’s in use half-life, fraction of degradable organic C that decomposes *DOC*_*F*_ = 0.1, and the CH_4_ generation rate *k*_*CH4*_ = 0.025. For paper HWP, the respective parameter values were *k*_*ret*_ = 0.2773 which corresponds to 2.5 years of HWP in use half-life, fraction of degradable organic C that decomposes *DOC*_*F*_ = 0.5, and the CH_4_ generation rate *k*_*CH4*_ = 0.05. We used *F* = 0.5 as the fraction of methane in generated emissions for both solid and paper HWP as recommended by IPCC [12]. The correction factor for aerobic decomposition in the year of disposal (*MCF* = 1.0) reflected the average values for Canada used in [4] which assumes all municipal solid waste landfills in Canada are anaerobically managed. Finally, the global warming potential of CH_4_, *GWP*_*100*_, was equal to 27.9 [7]. The complete list of parameter values is in Table S1.

Climate warming effect calculated in (10) is based on the mass balance approach that uses *GWP*_*100*_ to capture the effect on CH_4_ emissions released by decomposing HWP placed in the landfill. This approach does not reflect the temporal evolution of the effects of CH_4_ emissions on the climate system. To assess whether accounting for the temporal aspect of CH_4_ emissions would change the qualitative pattern of climate warming effect’s dependence on the proportional distribution of the disposed HWP among disposal options, we used the dynamic approach developed by Levasseur et al. [19]. This approach quantifies and sums up radiative forcing resulting from all CO_2_ and CH_4_ emissions attributable to disposal of the original HWP over any chosen time horizon. The formulae and results for this approach are presented in Supplementary Information.

The climate warming effect was estimated for 25, 50, 75, and 100 years from the year of disposal of the original HWP for scenarios corresponding to 0.1 incremental steps in the fractions allocated to landfilling *W*_*0*_ and recycling *R*_*0*_. For each scenario, the fraction allocated to burning was calculated from (1). We present results calculated over 100 years below, and those for 25, 50, and 75 years in Supplementary Information. In the main set of analyzed scenarios, the CH_4_ fraction o captured or flared in landfills, *Rec* = 0.42, was taken from [4]. It should be noted that *Rec* also includes the oxidized fraction of CH_4_ thus assuming the effect of oxidation factor *OX* on (3). To assess the role of the CH_4_ fraction captured, flared, or oxidized in landfills on the climate warming effect *C*_*Eq_total*_(*T*), the analysis was repeated for *Rec* values ranging from 0 to 1 at 0.05 increments.

## Results

**Fig. 2:**
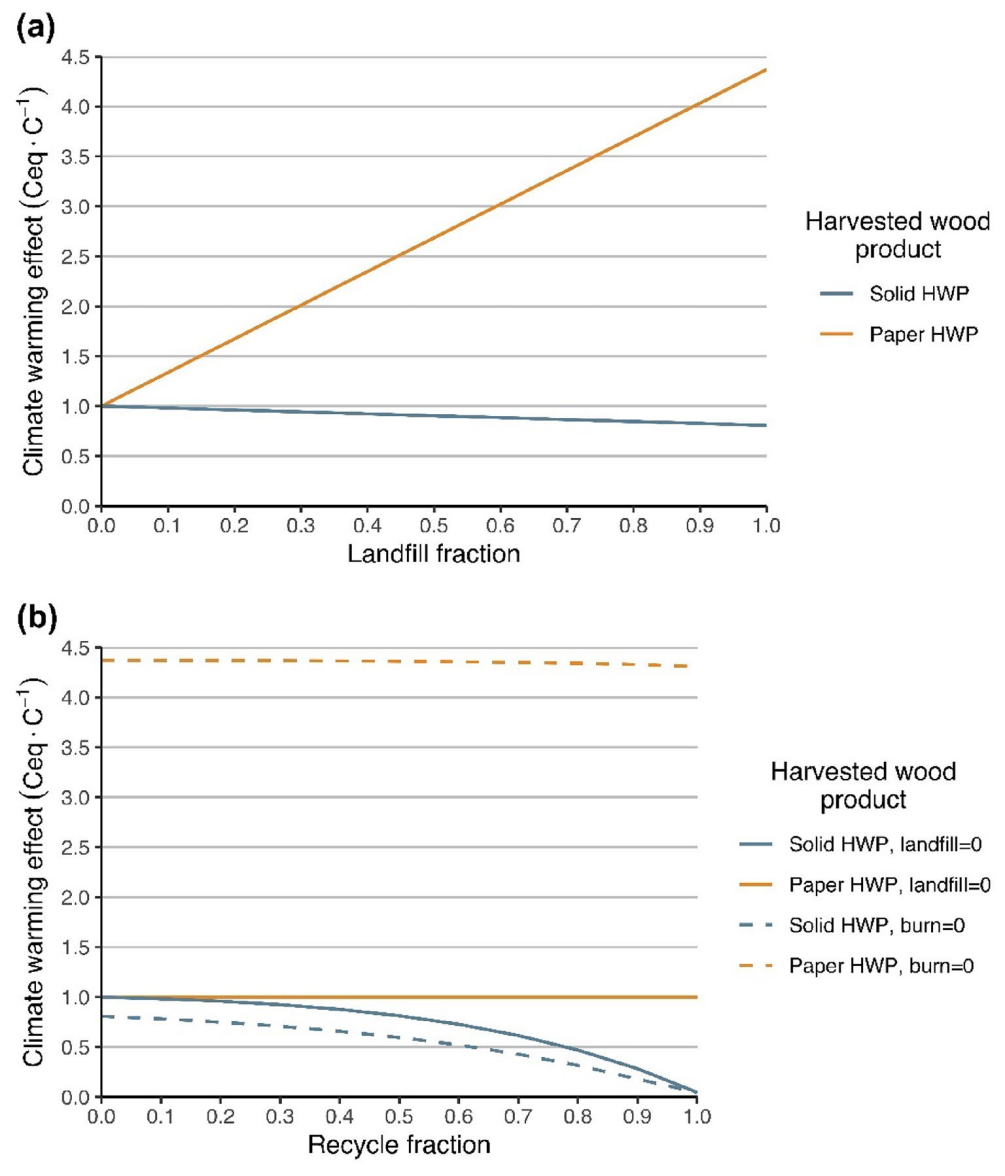
Climate warming effect of disposing of one unit of C in solid and paper HWP over 100 years, with the (**a**) recycle fraction set at zero, and (**b**) landfill (solid lines) or burn (dashed lines) fractions set at zero

The “extreme” scenarios correspond to one of the disposal fractions held constant at zero. The climate warming effect of disposing one unit of C in HWP calculated over 100 years is shown in Fig. 2; the effect for shorter periods of time (25, 50, and 75 years) is shown in Figure S2. As expected, the climate warming effect equals one when both landfill and recycling fractions equal zero, that is, when the entire unit of HWP is burned and emitted as CO_2_ (Fig. 2a). With the recycle fraction kept at zero, the climate warming effect of solid HWP decreases as the landfill fraction increases while the opposite is true for paper HWP (Fig. 2a), reflecting the difference in the decomposable fraction, *DOC*_*f*_, between solid and paper HWP.

**Fig. 3:**
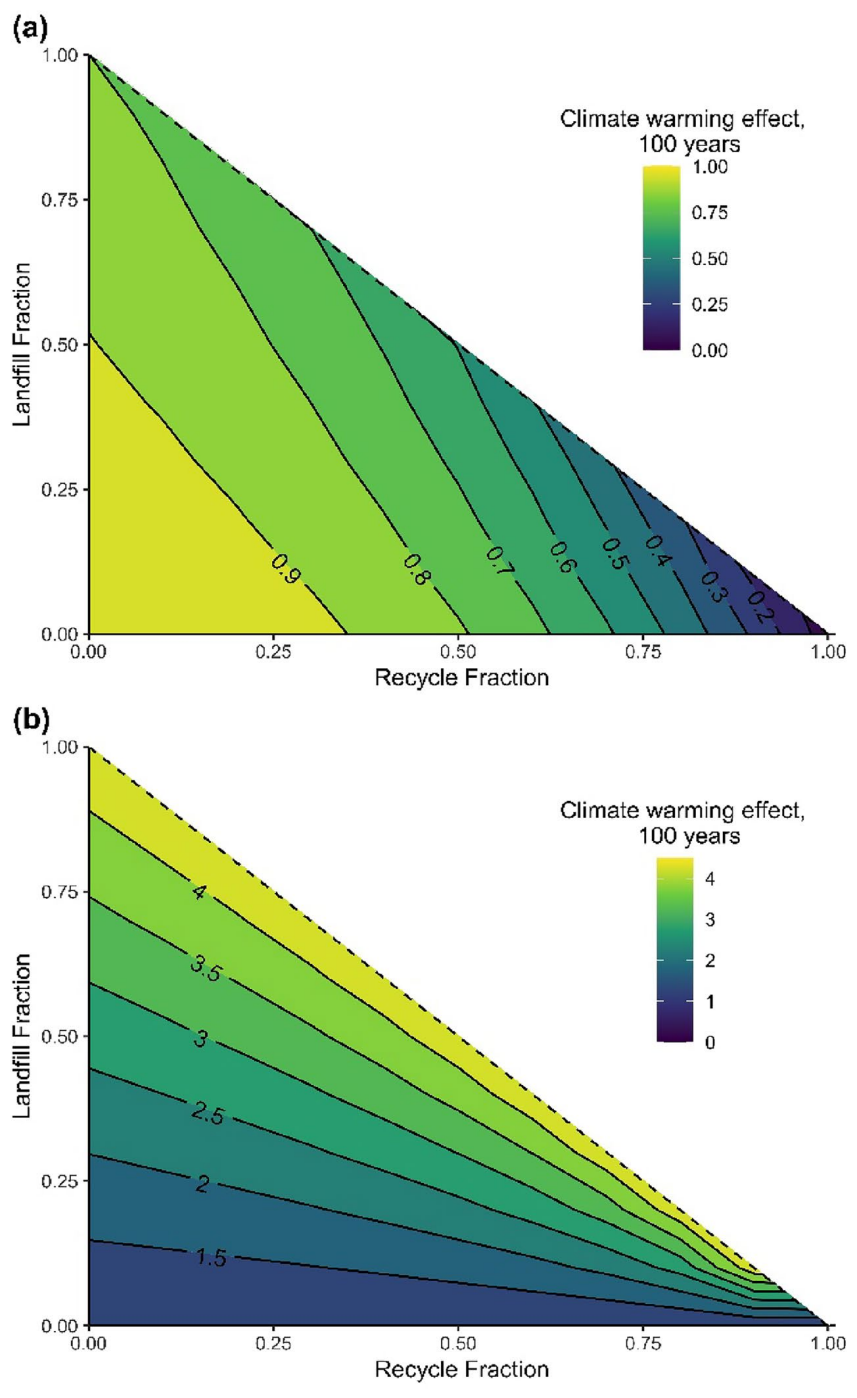
Relationship between the 100-year climate warming effect and recycle and landfill fractions for (**a**) solid and (**b**) paper HWP

Increasing the recycle fraction had negligible effects on climate warming effect of paper HWP over a 100-year period when burn or landfill fractions were held at zero (Fig. 2b); the short half-life of in use paper HWP leads to most of the disposed unit getting burned (when landfill fraction equals zero) or landfilled (when burned fraction equals zero) after going through the maximum number of recycling steps. For solid HWP, however, increasing recycling fraction keeps most of the original HWP unit in use longer, thus delaying the emissions beyond 100 years (Fig. 2b). The complete pattern of change in the climate warming effect calculated over 100 years with changes in recycle and landfill fractions for solid and paper HWP is shown in Fig. 3 with the burn fraction calculated as the difference between one and the sum of recycle and landfill fractions; similar complete patterns for climate warming effect calculated over shorter periods of time (25, 50, and 75 years) are shown in Figure S3.

**Fig. 4:**
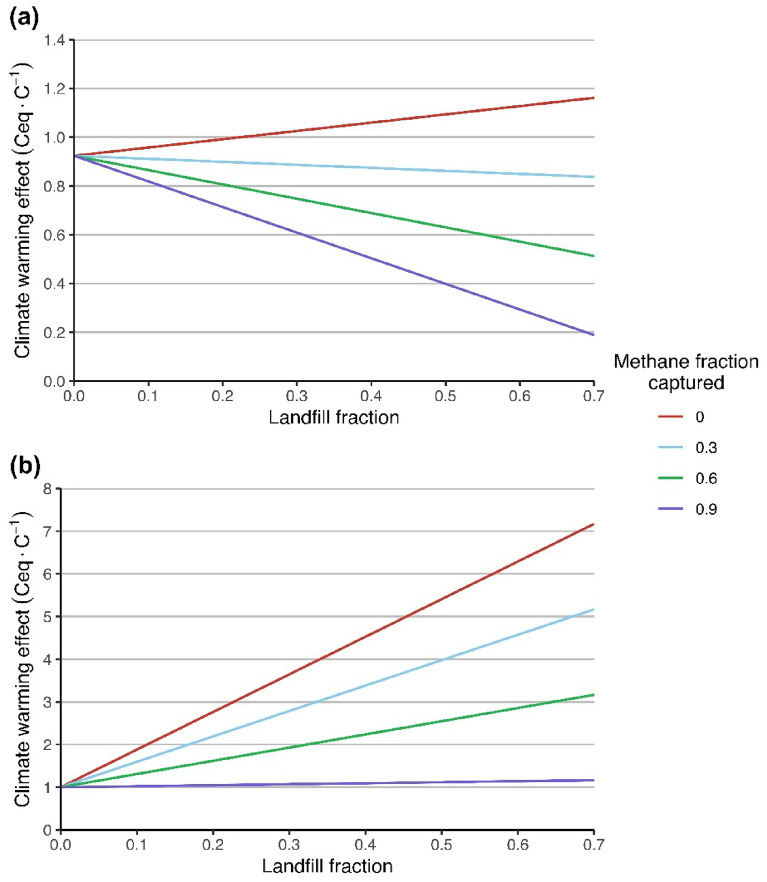
Changes in the 100-year climate warming effect with increases in the landfill fraction, with a recycle fraction of 0.3 and various methane capture rates in the landfill for (**a**) solid and (**b**) paper HWP

For any given recycling level, the climate warming effect for solid HWP decreases as the landfill fraction increases (Fig. 3a); this trend holds for various CH_4_ capture rates in landfills (*Rec*) unless the latter rate becomes close to 0.22. Conversely, the climate warming effect for paper HWP increases as landfill fraction increases unless the CH_4_ capture rate in landfills gets close to one. Changes in the climate warming effect in response to the changes in CH_4_ capture rate are the same for various fixed recycling fractions (Fig. 4 for the recycling fraction set at 0.3).

## Discussion

We estimated changes in the climate warming effect from disposing HWP containing one unit of C in response to the disposal fate fractions. For a given recycling level, increasing the fraction of solid HWP destined for landfills reduces the climate warming effect (Fig. 3a) because most of the product remains undecomposed for the 100 year period, effectively creating a long-term C sink [37, 38]. The opposite is true for paper HWP because more decomposes in the landfill (Fig. 3b). Since paper HWP decomposition occurs as CO_2_ and CH_4_, the climate warming effect increases, making incineration the preferred option for paper HWP.

These trends were established using default parameter values recommended in IPCC [12] and Canadian average rate of CH_4_ capture for energy or flaring [4]. However, these trends hold for a range of CH_4_ capture rates (Fig. 4). To reverse the trend for solid HWP, CH_4_ capture rates in landfills needs to be reduced to about 0.22. Similarly, the CH_4_ capture rate must increase to about 0.93 for the climate warming effect of paper HWP to decrease when landfill fraction increases. The role of CH_4_ capture rate was assessed because changes to this rate through waste management at landfills are likely more achievable than to the decomposable fractions of HWP and their rate of decomposition in anaerobic conditions. Note that default values for the latter parameters have been updated in IPCC [12] to reflect the most recent studies on HWP decomposition in landfills since the previously recommended values could have resulted in substantial overestimation of landfill emissions (e.g., [29]).

We found that recycling helps reduce the climate warming effect of solid HWP by keeping a larger fraction of products in use for a long period, effectively pushing some of the emissions beyond the study period. Increasing their recycled fraction while keeping the incineration fraction low is also better for reducing the climate warming effect than increasing the landfilled fraction (Fig. 3a). For example, recycling and landfilling fractions of 0.6 and 0.4, respectively, lowers climate warming effect more than fractions of 0.4 and 0.6, respectively. This result, however, depends on the CH_4_ capture rate in landfills and is likely to reverse if the CH_4_ capture rate in landfills increases. The emission-delaying effect of recycling on paper HWP can be seen only when the study period is short (e.g., Fig. S2d and S2f). As study length increases, paper HWP will be recycled their maximum allotted times, therefore being incinerated, or landfilled during the study period. Similar trends were observed in the scenario with the maximum number of recycling steps set at 2 and 7 for solid and paper HWP, respectively (Supplementary Information, Figures S4-S6); the climate warming effect of solid HWP remains below 1 despite the reduced number of recycling steps while with the increase the maximum number of recycling steps for paper HWP from 5 to 7 the emission delaying effect of recycling can be seen only for the short study periods: if assessed at 25 years from the original HWP disposal, the recycling rate must be higher than 0.9 to keep the climate warming effect of disposed paper HWP below one (Fig. S4f).

The relationship between the number of recycling steps and the length of study period and the consistency of the results between maximum recycling scenarios places less emphasis on the assumed maximum recycling steps used in this study. The number of recycling steps for paper HWP can vary from 5 and 7, after which the fibres become too short to create new paper [5, 35], although some studies suggest that paper HWP can be recycled substantially more than 5–7 times (e.g., [16]). The number of recycling steps for solid HWP is studied for cascading wood use that occurs when products are processed for at least one more use, either for material or energy purposes. In a single-stage cascade, wood is processed into a product that is used once more for energy purposes, while in a multi-stage cascade, wood is processed to be used at least once more in material form before disposal or recovery for energy purposes [36]. Multi-stage cascading appears to be limited to particleboards, and can include three (e.g., [22]), four (e.g., [10, 30]) or five to six cascading steps [8]. Multi-stage cascading, however, is bound by economic and technical considerations, with research and development efforts required to solve technical and logistical problems so cost-effective solutions can be realized [36].

The results presented in Figs. 1, 2, 3, 4 and S1-S6 were produced using the *GWP*_*100*_-based mass balance approach. *GWP*_*100*_ is defined as the time-integrated radiative forcing over 100 years due to a pulse emission of a given GHG relative to the pulse emission of an equal mass of CO_2_ [1]. Thus, using this approach effectively assumes that the 100-year greenhouse impact occurs in the same year as the pulse emission [9]. To test whether the estimated pattern in the climate warming effect at times shorter than 100 years from the date of the original HWP disposal is affected by *GWP*_*100*_-based approach, we estimated dynamic climate warming effect using dynamic characterization factors [19]. This approach quantifies for any given point in time the radiative forcing resulting from all CO_2_ and CH_4_ emissions that occurred prior to this point. The results corroborate the *GWP*_*100*_-based conclusions about the qualitative pattern of climate warming effect’s dependence on the proportional distribution of the HWP among disposal options (Supplementary Information, Figures S7-S9).

The results of our analysis are not directly comparable to those of many other studies on the post-use GHG emissions of HWP because we did not assign substitution effects to any of the disposal. Such substitution effects are expected to occur when products are incinerated for energy (either heat or electricity) and are usually assumed to be substantial. For example, in the meta-analysis of 51 studies, Leskinen et al. [17] estimated the average substitution factor from energy recovery at the end-of-life stage as 0.4 kg C/kg C wood product. However, recent review papers suggested that substitution effects may be frequently over-estimated [11, 18]. For incineration of HWP for heat, Villanueva and Wenzel [35] stated that it is difficult to ascribe the substitution benefits to the heat produced by incineration plants because it is often in excess of district heating needs. On the broader issue of displacing fossil fuels for electricity generation, York [39] concluded that “the average pattern across most nations of the world over the past fifty years is one where … each unit of electricity generated by non-fossil-fuel sources displaced less than one-tenth of a unit of fossil-fuel-generated electricity.”

The common assumptions about substitution effect of recycling are that wood is not used to produce virgin products, but rather energy (thus displacing the use of fossil fuels) or that the area harvested for HWP is reduced [24, 35]. Aside from questions about the effectiveness of displacing fossil fuels, the benefits of using wood for energy generation to mitigate climate change depend on many factors such as the choice of counterfactual scenario, the type of fossil fuel displaced, and the length of time over which the effects are estimated. For example, harvesting standing trees for energy can increase GHG emissions that can last decades or even centuries [18, 25] meaning that the substitution effect assigned to recycling is actually negative. Reducing harvest area because of recycling also does not automatically imply positive substitution effects since C stocks in forests saved from harvest may change due to succession or natural disturbances.

Some studies have also questioned climate change mitigation benefits of recycling, due to the energy required to recycle [34]. However, those concerns are not intended to dispute the overall benefits of recycling or incineration for energy [3, 21] but to highlight that quantifying their benefits to climate change mitigation is not simple. Our analysis side-stepped this ambiguity by focusing only on the climate warming effect of C contained in post-used HWP. We believe that this simplified approach offers useful insights into the role of HWP in GHG emissions. For example, it can be used to quickly assess the effects of the final disposal of recycled HWP when another recycling step is no longer an option. Results can also be used to assess whether, in reporting the GHG emissions (e.g., in the national GHG inventory reports), it is adequate to assume that the post-use HWP are simply emitted as CO_2_ or if this assumption over- or underestimates their effect on the climate system. Our results suggest that the answer depends on the composition of post-use HWP: for solid HWP, equating the post-use emissions with the HWP C content would underestimate their climate warming effect, while the opposite occurs for paper HWP, with these climate warming effects of solid and paper HWP holding true for a wide range of parameters values defined by disposal management.

## Conclusions

When analyzing how the climate warming effect of carbon content in disposed HWP changes with fractions destined for incineration, landfilling, and recycling, we found:

- For solid HWP, for any given recycling fraction the effect decreases as the landfill fraction decreases; consequently, the climate warming effect is lower when less solid HWP is incinerated
- For paper HWP, increasing the incinerated HWP fraction reduces the climate warming effect
- For solid HWP, recycling reduces climate warming by “pushing” some of the emissions outside of the assessment period; while for paper HWP, recycling does not change the climate warming effect unless the assessment period is relatively short
- The estimated climate warming effects are driven primarily by the decomposable fractions and the rate of anaerobic HWP decomposition in landfills; opposite patterns are observed if the CH_4_ capture rate is very low for solid HWP or very high for paper HWP.

The results were produced without assigning possible substitution effects to incineration or recycling. The analysis was focused strictly on the climate warming effect of carbon content in disposed HWP with no consideration for other logistical, environmental, and health hazard aspects of waste disposal.

## Supplementary Information

Additional file 1.

## Data Availability

No datasets were generated or analysed during the current study.
