# Supplementary material for: Climate warming effect of disposal fates of harvested wood products
Source: Carbon Balance Manag. 2026 Apr 21;21:87. doi: 10.1186/s13021-026-00442-4 (PMC13289483; doi:10.1186/s13021-026-00442-4)
Supplement: Supplementary file 1 — Additional file 1. [file 13021_2026_442_MOESM1_ESM.docx]

**Climate warming effect of disposal fates of harvested wood products – Supplementary Information**

Michael T. Ter-Mikaelian^1^, Sabrina M. Desjardins^1^, and Jiaxin Chen^1^

^1^ Ontario Ministry of Natural Resources, Ontario Forest Research Institute, 1235 Queen St. East, Sault Ste. Marie, Ontario P6A 2E5

Table S1. Parameter values used in the analysis of climate warming effects of solid and paper HWPs.

| **Parameter** | **Value** | **Source** |
| --- | --- | --- |
| HWP in use |  |  |
| Solid HWP |  |  |
| Half-life (years) | 35 | IPCC 2019 |
| Retirement rate, *k_ret_* (year^-1^) | 0.0198 | IPCC 2019 |
| Paper HWP |  |  |
| Half-life (years) | 2.5 | IPCC 2019 |
| Retirement rate, *k_ret_* (year^-1^) | 0.2773 | IPCC 2019 |
| Fraction of degradable carbon that decomposes in landfills, *DOC_f_* |  |  |
| Solid HWP | 0.1 | IPCC 2019 |
| Paper HWP | 0.5 | IPCC 2019 |
| Methane generation rate, *k_CH4_* (year^-1^)^a^ |  |  |
| Solid HWP | 0.025 | IPCC 2019 |
| Paper HWP | 0.05 | IPCC 2019 |
| Fraction of methane in emissions generated in landfills, *F* |  |  |
| Solid and paper HWP | 0.5 | IPCC 2019 |
| Methane correction factor, *MCF* | 1.0 | ECCC 2024 |
| Fraction of methane captured or flared in landfills, *Rec*^b^ | 0.42 | ECCC 2024 |
| Global warming potential of methane, *GWP_100_* | 27.9 | Forster et al. 2021 |

^a^ The mean of default values for dry and wet conditions in Boreal and Temperate Climate Zone

^b^ Includes fraction of methane oxidised by cover layers, OX

Figure S1. Transfers of carbon during the first three years from the original (virgin) HWP disposal. Here *B*(*n*) is the amount of HWP burned in year *n*, *W*(*n*) is the amount of HWP placed in the landfill in year *n*, *R*(*n*) is the total amount of HWP recycled in year *n*, *E_CO2_*(0|*B*(*n*)) are CO_2_ emissions from burning HWP in year *n*, and *E_CO2_*(*m*|*W*(*n*)) and *E_CH4_*(*m*|*W*(*n*)) are landfill CO_2_ and CH_4_ emissions, respectively, *m* years after *W*(*n*) of HWP is placed in the landfill in year *n.*


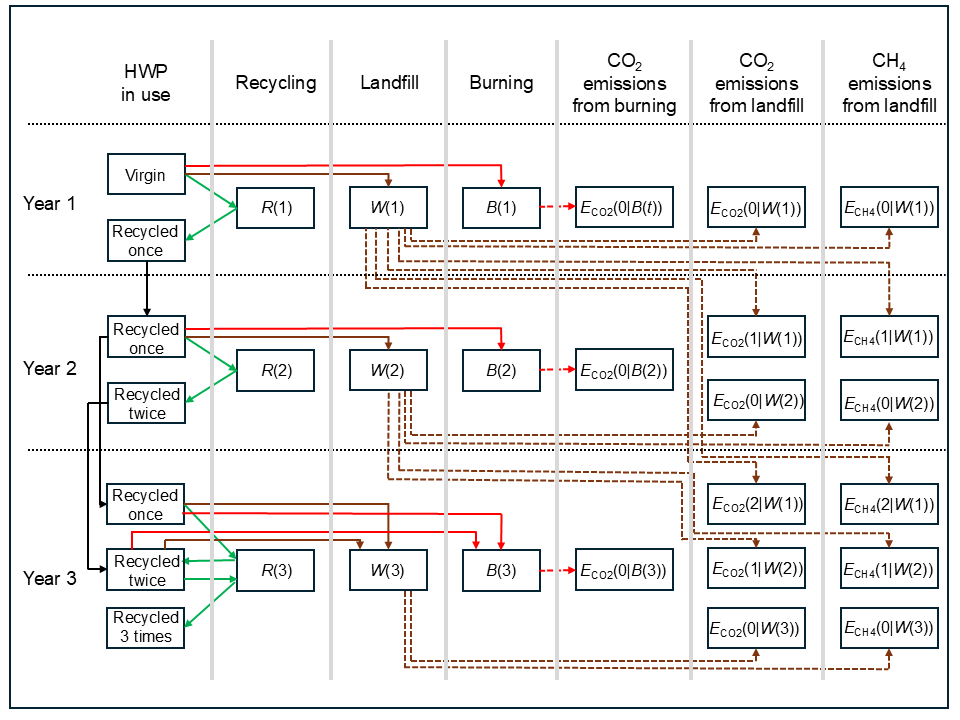


Figure S2. GWP_100_-based climate warming effect of disposing of one unit of C in solid (a, c, e) and paper (b, d, f) HWP over 25 (red lines), 50 (light blue lines), 75 (green lines) and 100 (purple lines) years, with the (a, b) recycle fraction set at zero, (c, d) landfill fraction set at zero and (e, f) burn fraction set at zero. The maximum number of recycling steps is equal to 5 for both solid and paper HWP.


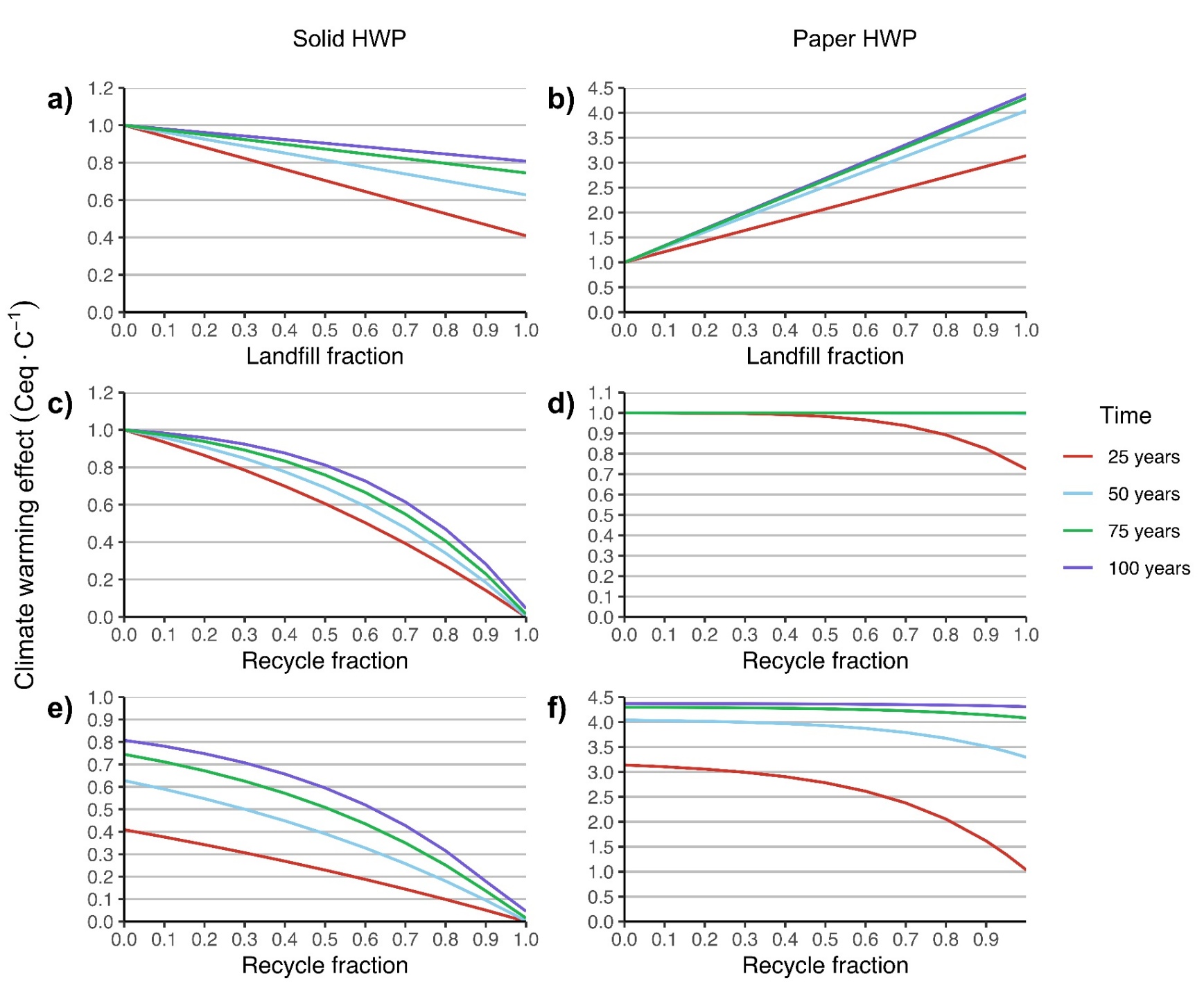


Figure S3. Relationship between the (a, d) 25-year, (b, e) 50-year, and (c, f) 75-year GWP_100_-based climate warming effect and recycle and landfill fractions for (a, b, c) solid and (d, e, f) paper HWP. The maximum number of recycling steps is equal to 5 for both solid and paper HWP.


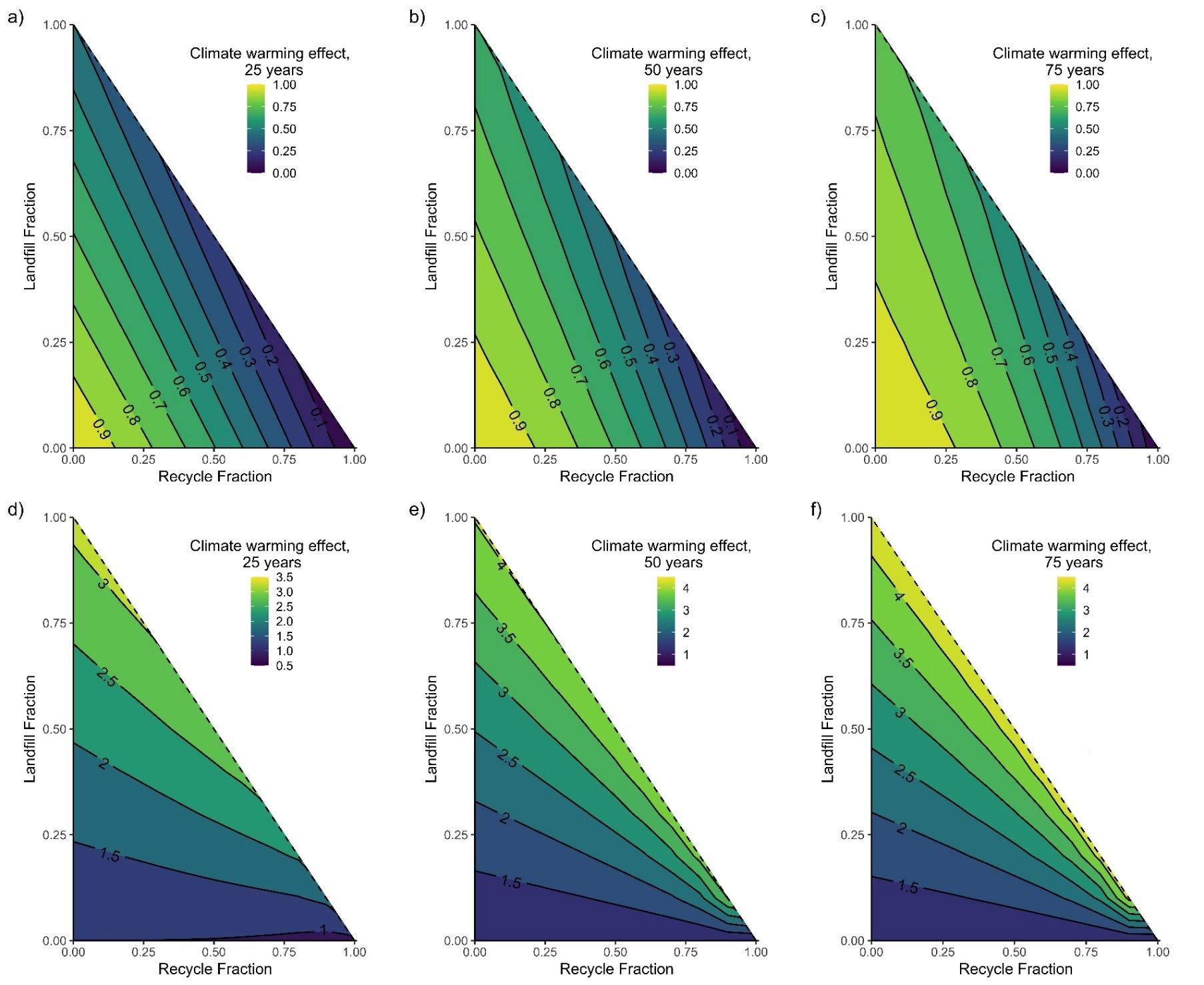


**Alternative maximum number of recycling steps**

In this section we estimate the climate warming effect of the disposed HWP for the alternative maximum number of recycling steps that a virgin product can undergo after being retired out of use, namely: 2 and 7 recycling steps for solid and paper HWP, respectively. Derivation of the formulae used to calculate fractions of the product placed in the landfill and burned in year *n* follows the same logic as that used for formulae (6-9) in the main text. We consider all possible sequences of events that lead to the fraction of product being landfilled or burned in a given year. The general formula for the fraction of the original unit of solid HWP landfilled in year *n* is:

| (S1) | ${W\left( n \right)=Ret\left( n-1 \right)\cdot R_{0}\cdot W}_{0}+\sum_{l_{1}=1}^{\left( n-1 \right)-1} Ret\left( l_{1} \right)\cdot Ret\left( n-1-l_{1} \right)\cdot R_{0}^{2}\cdot W_{0}/(B_{0}+W_{0})$ |
| --- | --- |

where *Ret*(*l*) is the fraction of HWP retired *l* years after its production. Using denotation *S* = exp( - *k_ret_*) where *k_ret_* is the product retirement rate, formula (S1) can be re-written as:

| (S2) | $W\left( n \right)=S^{n-2}\cdot\left( 1-S \right)\cdot R_{0}\cdot W_{0}+\binom{n-2}{1}\cdot S^{n-3}\cdot\left( 1-S \right)^{2}\cdot R_{0}^{2}\cdot W_{0}/(B_{0}+W_{0)}$ |
| --- | --- |

Formula (S2) applies to *n* > 2; for *n* = 1, 2 it simplifies to

| (S3) | $W\left( 2 \right)=\left( 1-S \right)\cdot R_{0}\cdot W_{0}; W\left( 1 \right)=W_{0}$ |
| --- | --- |

For paper HWP, general formula for the fraction of the original unit of HWP landfilled in year *n* is:

| (S4) | ${W\left( n \right)=Ret\left( n-1 \right)\cdot R_{0}\cdot W}_{0}+\sum_{l_{1}=1}^{\left( n-1 \right)-1} Ret\left( l_{1} \right)\cdot Ret\left( n-1-l_{1} \right)\cdot R_{0}^{2}\cdot W_{0}+$ $\sum_{l_{1}=1}^{\left( n-1 \right)-2} \sum_{l_{2}=1}^{\left( n-1 \right)-1-l_{1}} Ret\left( l_{1} \right)\cdot Ret\left( l_{2} \right)\cdot Ret\left( n-1-l_{1}-l_{2} \right)\cdot R_{0}^{3}\cdot W_{0}+$ $\sum_{l_{1}=1}^{\left( n-1 \right)-3} \sum_{l_{2}=1}^{\left( n-1 \right)-2-l_{1}} \sum_{l_{3}=1}^{\left( n-1 \right)-1-l_{1}-l_{2}} Ret\left( l_{1} \right)\cdot Ret\left( l_{2} \right)\cdot Ret\left( l_{3} \right)\cdot Ret\left( n-1-l_{1}-l_{2}-l_{3} \right)\cdot R_{0}^{4}\cdot W_{0}+$  $\sum_{l_{1}=1}^{\left( n-1 \right)-4} \sum_{l_{2}=1}^{\left( n-1 \right)-3-l_{1}} \sum_{l_{3}=1}^{\left( n-1 \right)-2-l_{1}-l_{2}} \sum_{l_{4}=1}^{\left( n-1 \right)-1-l_{1}-l_{2}-l_{3}} Ret(l_{1})\cdot Ret(l_{2})\cdot Ret(l_{3})\cdot Ret(l_{4})\cdot{Ret(n-1-l_{1}-l_{2}-l_{3}-l_{4})\cdot R}_{0}^{5}\cdot W_{0}+$  $\sum_{l_{1}=1}^{\left( n-1 \right)-5} \sum_{l_{2}=1}^{\left( n-1 \right)-4-l_{1}} \sum_{l_{3}=1}^{\left( n-1 \right)-3-l_{1}-l_{2}} \sum_{l_{4}=1}^{\left( n-1 \right)-2-l_{1}-l_{2}-l_{3}} \sum_{l_{5}=1}^{\left( n-1 \right)-1-l_{1}-l_{2}-l_{3}-l_{4}} Ret(l_{1})\cdot Ret(l_{2})\cdot Ret(l_{3})\cdot Ret(l_{4})\cdot{Ret(l_{5})\cdot Ret(n-1-l_{1}-l_{2}-l_{3}-l_{4}-l_{5})\cdot R}_{0}^{6}\cdot W_{0}+$  $\sum_{l_{1}=1}^{\left( n-1 \right)-6} \sum_{l_{2}=1}^{\left( n-1 \right)-5-l_{1}} \sum_{l_{3}=1}^{\left( n-1 \right)-4-l_{1}-l_{2}} \sum_{l_{4}=1}^{\left( n-1 \right)-3-l_{1}-l_{2}-l_{3}} \sum_{l_{5}=1}^{\left( n-1 \right)-2-l_{1}-l_{2}-l_{3}-l_{4}} \sum_{l_{6}=1}^{\left( n-1 \right)-1-l_{1}-l_{2}-l_{3}-l_{4}-l_{5}} Ret(l_{1})\cdot Ret(l_{2})\cdot Ret(l_{3})\cdot Ret(l_{4})\cdot Ret(l_{5})\cdot Ret(l_{6})\cdot Ret(n-1-l_{1}-l_{2}-l_{3}-l_{4}-l_{5}-l_{6})\cdot R_{0}^{7}\cdot W_{0}/(B_{0}+W_{0)}$ |
| --- | --- |

or using denotation *S* = exp( - *k_ret_*) where *k_ret_* is the product retirement rate

| (S5) | $W\left( n \right)=S^{n-2}\cdot\left( 1-S \right)\cdot R_{0}\cdot W_{0}+\binom{n-2}{1}\cdot S^{n-3}\cdot\left( 1-S \right)^{2}\cdot R_{0}^{2}\cdot W_{0}+$ $\binom{n-2}{2}\cdot S^{n-4}\cdot\left( 1-S \right)^{3}\cdot R_{0}^{3}\cdot W_{0}+\binom{n-2}{3}\cdot S^{n-5}\cdot\left( 1-S \right)^{4}\cdot R_{0}^{4}\cdot W_{0}+$  $\binom{n-2}{4}\cdot S^{n-6}\cdot\left( 1-S \right)^{5}\cdot R_{0}^{5}\cdot W_{0}+\binom{n-2}{5}\cdot S^{n-7}\cdot\left( 1-S \right)^{6}\cdot R_{0}^{6}\cdot W_{0}+$ $\binom{n-2}{6}\cdot S^{n-8}\cdot\left( 1-S \right)^{7}\cdot R_{0}^{7}\cdot W_{0}/(B_{0}+W_{0})$ |
| --- | --- |

Formula (S5) applies to *n* > 7; for *n* = 1, …,7. It simplifies to

| (S6) | $W\left( n \right)=(1-S)\cdot{((1-S)\cdot R_{0}+S)}^{n-2}\cdot R_{0}\cdot W_{0}$ |
| --- | --- |

For *n* = 1, *W*(1) = *W_0_*. For the formulae describing the fraction of HWP burned in year *n*, *B*(*n*), all instances of *W_0_* are replaced with *B_0_* in (S1-S2) and (S4-S5) except for the denominator in the last term in (S3) and (S6).Calculated fractions of *W* and *B* are substituted in formula (10) from the main text to estimate the climate warming effect of solid and paper HWP with the alternative maximum number of recycling steps.

For the ease of comparing the results of scenarios with different maximum number of recycling steps, climate warming effect of disposing of one unit of C with the maximum recycling steps set at 2 and 7 for solid and paper HWP, respectively, was estimated at 25, 50,75, and 100 years from the disposal of the original HWP. As with the results in the main text, climate warming effect is presented for the “extreme” scenarios corresponding to one of the disposal fractions held constant at zero (Figure S4) and for all possible combinations of recycle and landfill fractions (with the burn fraction calculated as the difference between one and the sum of recycle and landfill fractions) (Figures S5-S6).

Figure S4. GWP_100_-based climate warming effect of disposing of one unit of C in solid (a, c, e) and paper (b, d, f) HWP over 25 (red lines), 50 (light blue lines), 75 (green lines) and 100 (purple lines) years, with the (a, b) recycle fraction set at zero, (c, d) landfill fraction set at zero and ( e, f) burn fraction set at zero. The maximum number of recycling steps is equal to 2 and 7 for solid and paper HWP, respectively.


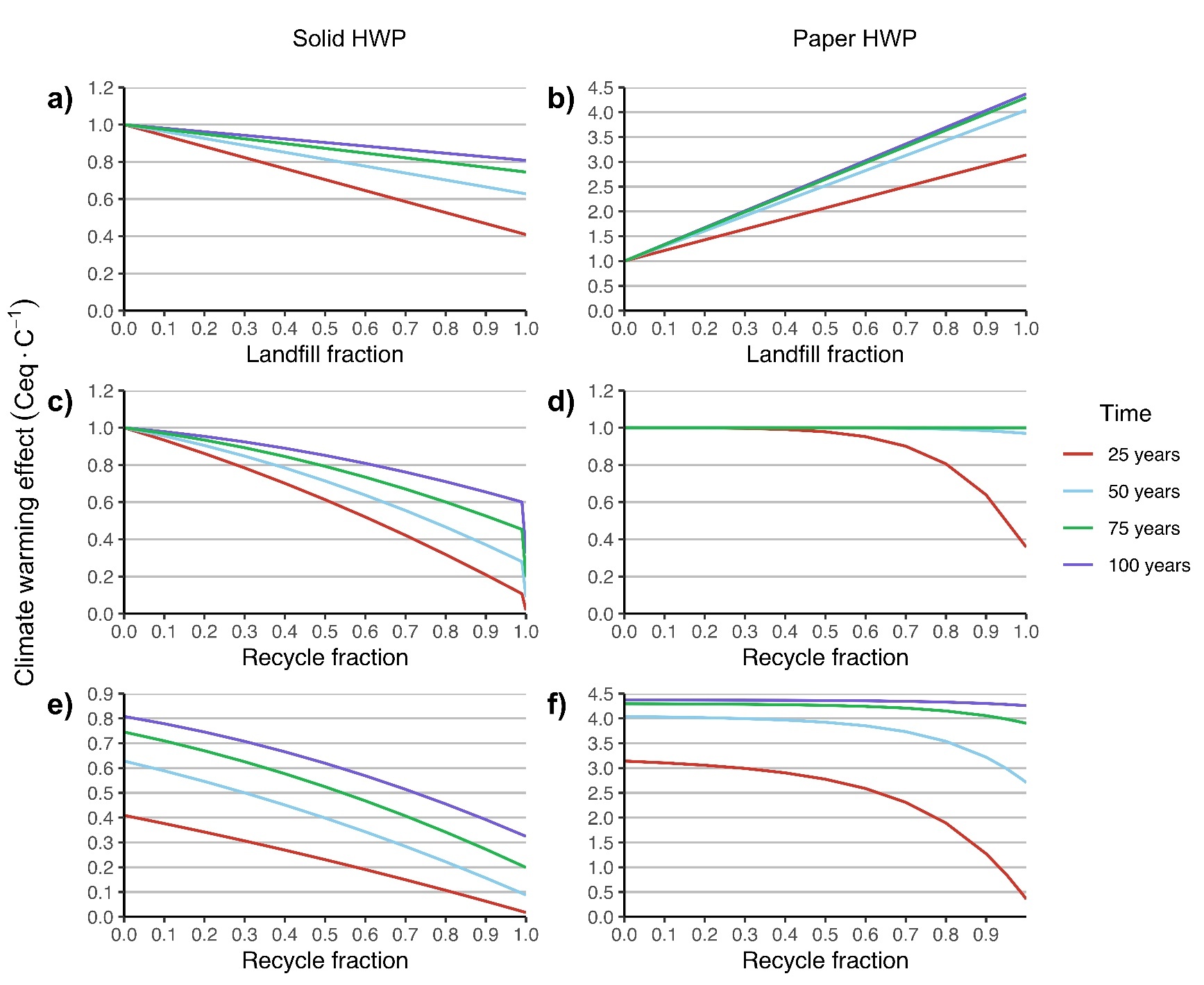


Figure S5. Relationship between the (a) 25-year, (b) 50-year, (c) 75-year, and (d) 100-year GWP_100_-based climate warming effect and recycle and landfill fractions for solid HWP. The maximum number of recycling steps for solid HWP is equal to 2.


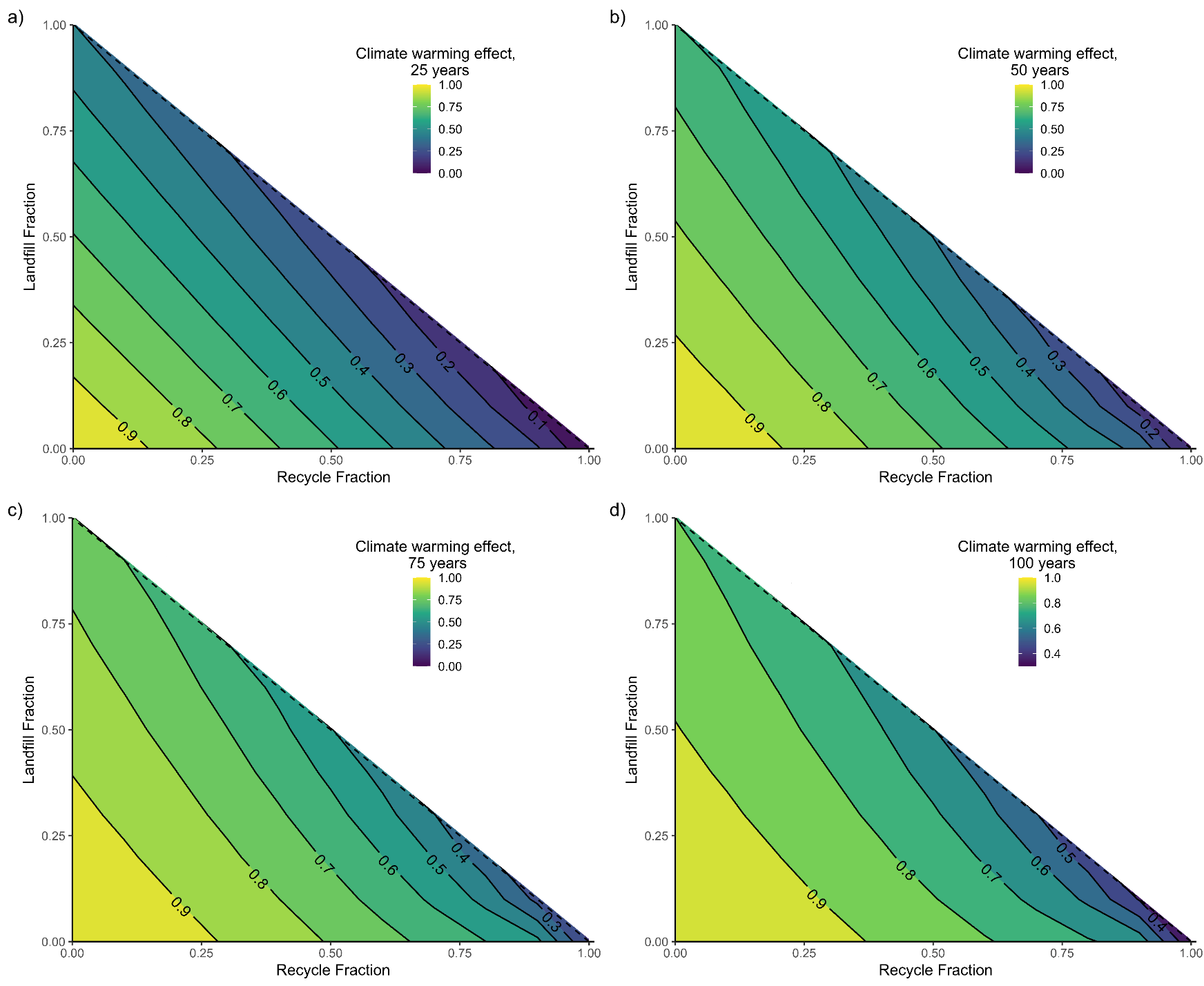


Figure S6. Relationship between the (a) 25-year, (b) 50-year, (c) 75-year, and (d) 100-year GWP_100_-based climate warming effect and recycle and landfill fractions for paper HWP. The maximum number of recycling steps for paper HWP is equal to 7.


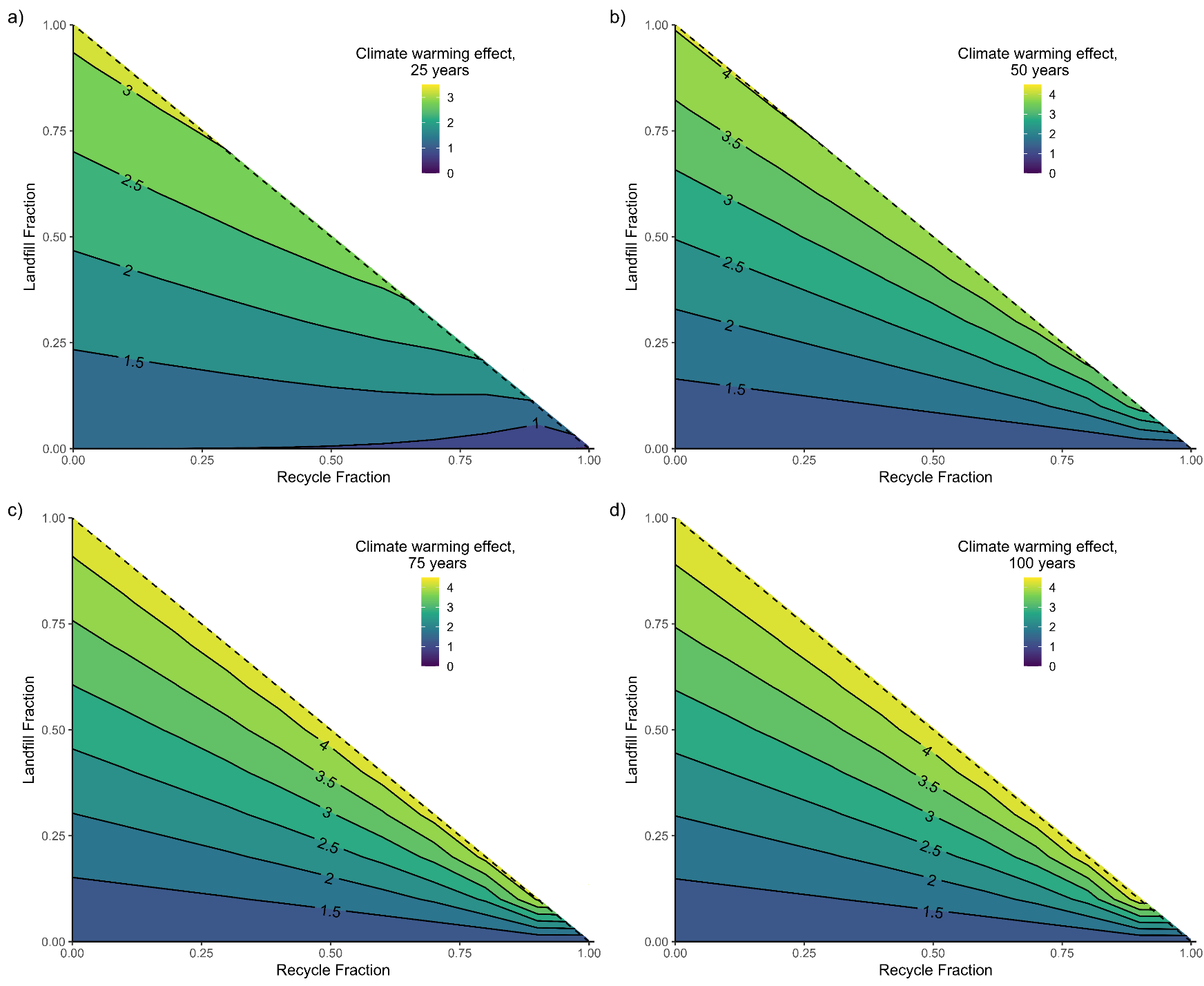


**Dynamic climate warming effect**

Calculation of the dynamic climate warming effect follows the methodology by (Levasseur et al. 2010) who developed instantaneous dynamic characterization factors (*DCF*) for global warming impact assessment of GHG emissions. Levasseur et al. (2010) defined instantaneous DCF for the time interval (*m*-1,*m*) after emission of a given gas *i* at time 0 as

| (S7) | ${DCF}_{i}(m)=\int_{m-1}^{m} {IRF}_{i}\cdot Q_{i}\left( m \right)dm$ |
| --- | --- |

where *IRF_i_* is the instantaneous radiative forcing per unit mass increase of gas *i* in the atmosphere and *Q_i_*(*t*) is the time-dependent atmospheric load of the released gas *i*; two gases relevant to our analysis are carbon dioxide (CO_2_) and methane (CH_4_). Instantaneous global warming impact (*GWI_inst_*) at time *t* of all releases of the two GHGs is calculated as

| (S8) | ${GWI}_{inst}(t)=\sum_{j=0}^{t} \left( g_{{CO}_{2}}\left( j \right)\cdot{DCF}_{{CO}_{2}}\left( t-j \right)+g_{{CH}_{4}}\left( j \right)\cdot{DCF}_{{CH}_{4}}\left( t-j \right) \right)$ |
| --- | --- |

where g_CO2_(j) and g_CH4_(j) are emissions of CO_2_ and CH_4_ at time j. In other words, global warming effect of gas *i* at time *t* reflects radiative forcing effect of all emissions of gas *i* that occurred from time 0 to *t*. Consequently, cumulative global warming impact (*GWI_cum_*) at time *T* is the sum of all instantaneous global warming impacts between 0 and *T*

| (S9) | ${GWI}_{cum}(T)=\sum_{t=0}^{T} {GWI}_{inst}(t)$ |
| --- | --- |

For CO_2_, the atmospheric load *Q_CO2_*(*t*) following a pulse emission is given by Bern carbon cycle-climate model

| (S10) | $Q_{CO2}\left( t \right)=a_{0}+\sum_{i=1}^{3} a_{i}\cdot exp\left( -\frac{t}{\tau_{i}} \right)$ |
| --- | --- |

where *a_0_* = 0.2173, *a_1_* = 0.2240, *a_2_* = 0.2824, *a_3_* = 0.2763, *τ_1_* = 394.4 years, *τ_2_* = 36.54 years, *τ_3_* = 4.304 years (Joos et al. 2013). For CH_4_, the atmospheric load *Q*(*t*) following a pulse emission is given by a first-order decay equation where the inverse of the kinetic constant is the adjusted lifetime *τ_CH4_*

| (S11) | $Q\left( t \right)=exp\left( -\frac{t}{\tau_{CH4}} \right)$ |
| --- | --- |

where *τ_CH4_* = 11.8 years (Forster et al. 2021).

Emissions in year *t* from the HWP disposal are given by formula (2) in the main text. Combining (2) with (S8) gives us the following formula for the instantaneous global warming impact at time *t*

| (S12) | ${GWI}_{inst}\left( t \right)={\sum_{n=1}^{t} E_{CO2}(0\vert B\left( n \right))\cdot{DCF}_{CO2}(t+1-n)+}$  $\sum_{n=1}^{t} \sum_{m=0}^{t-n} \left( E_{CO2}(m\vert W\left( n \right))\cdot{DCF}_{CO2}\left( t+1-n-m \right)+E_{CH4}(m\vert W\left( n \right))\cdot{DCF}_{CH4}(t+1-n-m) \right)$ |
| --- | --- |

Here *E_CO2_*(*m*|*W*(*n*)) and *E_CH4_*(*m*|*W*(*n*)) denote CO_2_ and CH4 emissions, respectively, *m* years after the amount *W*(*n*) of HWP is placed in the landfill in year *n*. Thus, instantaneous global warming impact at year *t* accounts for the radiative forcing effect of all emissions that occurred prior to year *t*. When the HWP is retired in year *n*, the *B*(*n*) fraction of it is incinerated generating CO_2_ emissions in year *n*; the effect of these emissions is quantified in the first term on the right-hand side of (S12). Another fraction *W*(*n*) of the HWP retired in year *n* is placed in the landfill. As this fraction decomposes, it keeps emitting both CO_2_ and CH_4_ in each year following *n*; the effects of each one of these emissions are captured in the second and third terms on the right-hand side of (S12).

The dynamic climate warming effect *C_Dyn_* at time *T* of disposing one unit of C in HWP in year 0 is calculated as the ratio of the cumulative global warming impact of emissions resulting from the disposal pathways (incineration, recycling, and landfilling) to that of the single pulse of emissions in which the entire unit of C is released as CO2 in year 0

| (S13) | $C_{Dyn}\left( T \right)=\frac{\sum_{t=0}^{T} {GWI}_{inst}(t as per formula \left( S12 \right))}{\sum_{t=0}^{T} {GWI}_{inst}(t from a signle pulse of {CO}_{2} at tme 0)}$ |
| --- | --- |

The ratio in (S13) allows for an easy interpretation of *C_Dyn_*(*T*): if it is less than 1 then the climate warming effect of the disposed HWP is less than if its entire C content was emitted as CO_2_, and vice versa. For consistency with the climate warming effect estimated using GWP_100_ (formula (10) in the main text), we present the results for *C_Dyn_*(*T*) at *T* = 25, 50, 75, and 100. Estimated dynamic climate warming effects for the “extreme” scenarios corresponding to one of the disposal fractions held constant at zero are shown in (Figure S7) and for various fractions of the disposed HWP going to incineration, recycling, and landfilling in Figures S8-S9.

Figure S7. Dynamic climate warming effect of disposing of one unit of C in solid (a, c, e) and paper (b, d, f) HWP over 25 (red lines), 50 (light blue lines), 75 (green lines) and 100 (purple lines) years, with the (a, b) recycle fraction set at zero, (c, d) landfill fraction set at zero and ( e, f) burn fraction set at zero. The maximum number of recycling steps is equal to 5 for both solid and paper HWP.


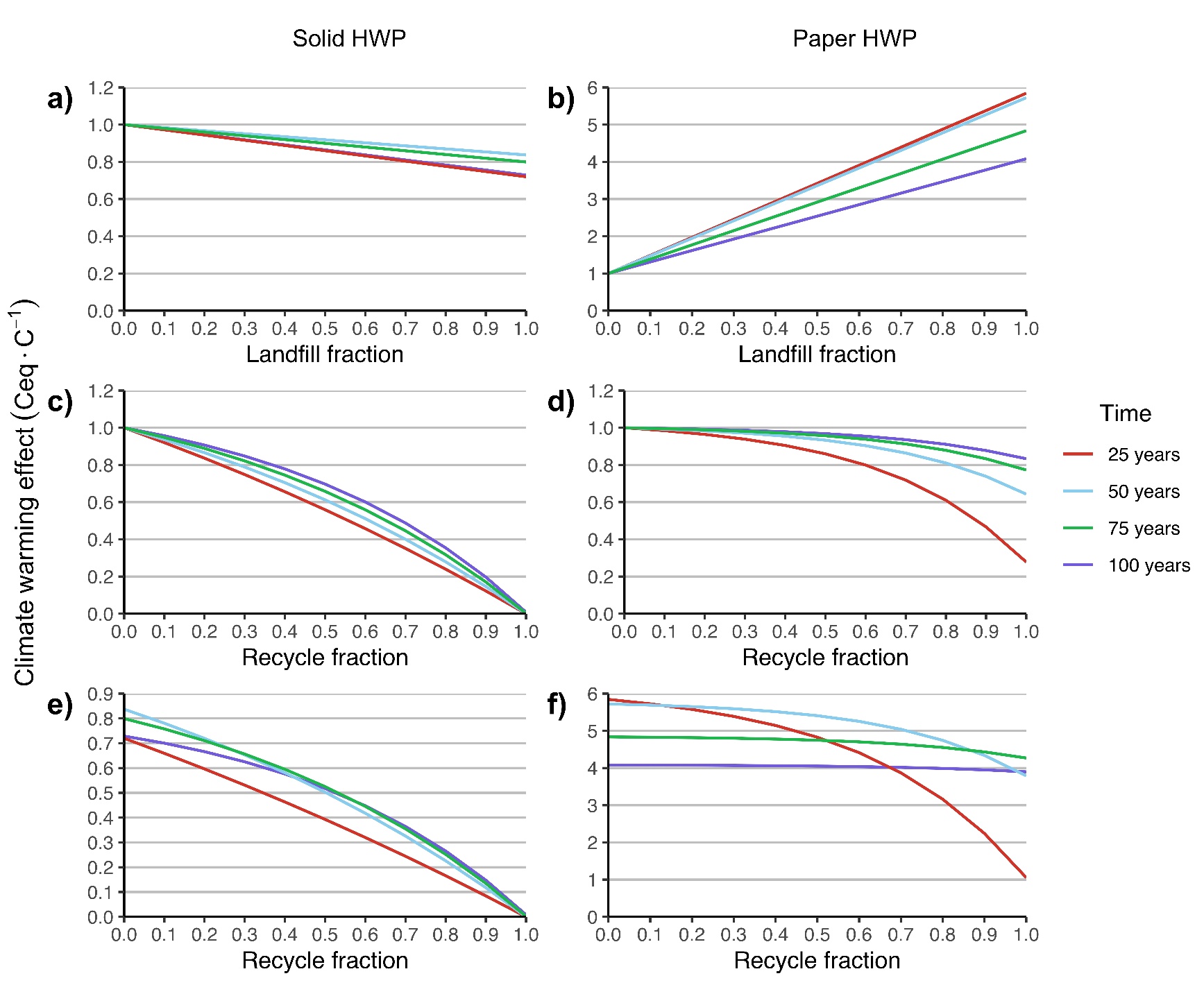


Figure S8. Relationship between the (a) 25-year, (b) 50-year, (c) 75-year and (d) 100-year dynamic climate warming effect and recycle and landfill fractions for solid HWP. The maximum number of recycling steps for solid HWP is equal to 5.


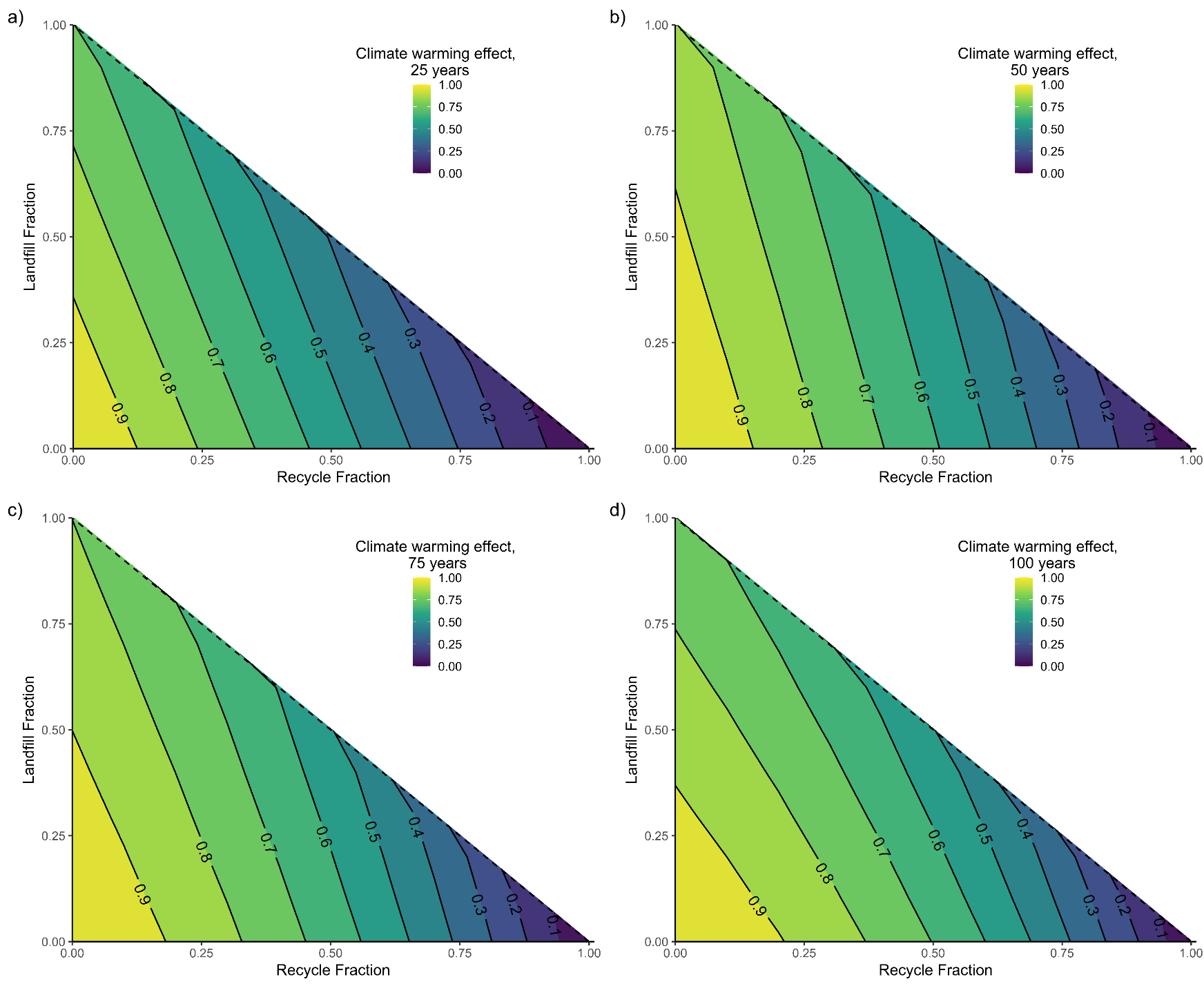


Figure S9. Relationship between the (a) 25-year, (b) 50-year, (c) 75-year and (d) 100-year dynamic climate warming effect and recycle and landfill fractions for paper HWP. The maximum number of recycling steps for paper HWP is equal to 5.


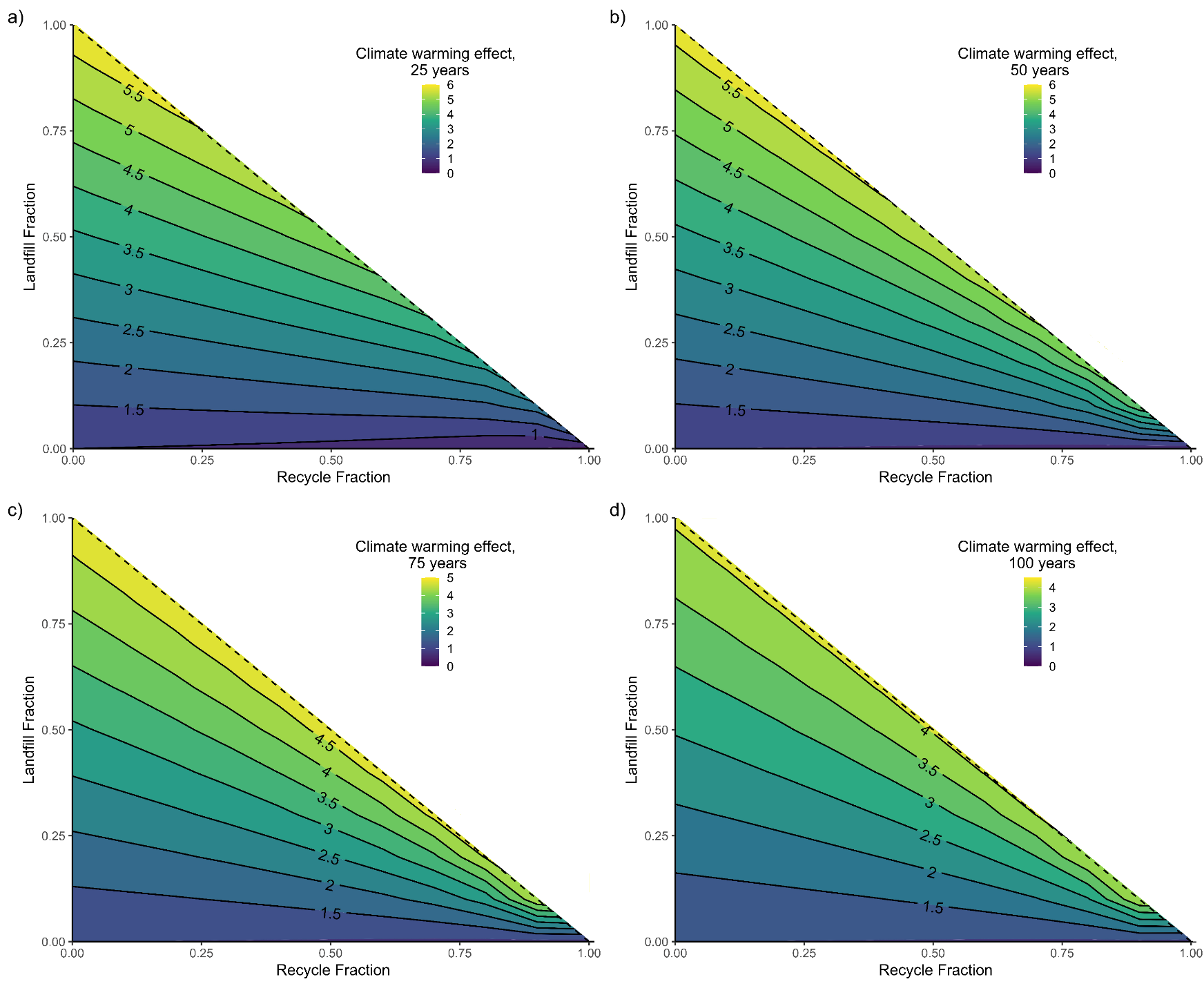


**References**

ECCC 2024. [Environment and Climate Change Canada]. 2024. National Inventory Report, 1990–2022: Greenhouse Gas Sources and Sinks in Canada. Available online at: https://unfccc.int/documents/638317. Accessed 20 March 2025.

Forster, P., Storelvmo, T., Armour, K., Collins, W., Dufresne, J.-L., Frame, D., Lunt, D.J., Mauritsen, T., Palmer, M.D., Watanabe, M., Wild, M., Zhang, H., 2021. The Earth’s energy budget, climate feedbacks, and climate sensitivity. In Climate change 2021: The physical science basis. Contribution of Working Group I to the Sixth Assessment Report of the Intergovernmental Panel on Climate Change [Masson-Delmotte, V., P. Zhai, A. Pirani, S.L. Connors, C. Péan, S. Berger, N. Caud, Y. Chen, L. Goldfarb, M.I. Gomis, M. Huang, K. Leitzell, E. Lonnoy, J.B.R. Matthews, T.K. Maycock, T. Waterfield, O. Yelekçi,

R. Yu, and B. Zhou (eds.)]. Cambridge University Press, Cambridge, United Kingdom and New York, NY, USA.

IPCC 2019. [Intergovernmental Panel on Climate Change]. 2019 Refinement to the 2006 IPCC Guidelines for National Greenhouse Gas Inventories, Calvo Buendia, E., Tanabe, K., Kranjc, A., Baasansuren, J., Fukuda, M., Ngarize, S., Osako, A., Pyrozhenko, Y., Shermanau, P. and Federici, S. (eds). Published: IPCC, Switzerland.

Joos, F., Roth, R., Fuglestvedt, J.S., Peters, G.P., Enting, I.G., Von Bloh, W., Brovkin, V., Burke, E.J., Eby, M., Edwards, N.R. Friedrich, T., Fr¨olicher, T. L., Halloran, T. L., Holden, T. L., Jones, C., Kleinen, T., Mackenzie, F. T., Matsumoto, F. T., Meinshausen, M., Plattner, G.-K, Reisinger, A., Segschneider, J., Shaffer, G., Steinacher, M., Strassmann, K., Tanaka, K., Timmermann, A., and Weaver A. J., 2013. Carbon dioxide and climate impulse response functions for the computation of greenhouse gas metrics: a multi-model analysis. Atmospheric Chemistry and Physics, 13(5), pp.2793-2825.

Levasseur, A., Lesage, P., Margni, M., Deschênes, L. and Samson, R., 2010. Considering time in LCA: dynamic LCA and its application to global warming impact assessments. Environmental science & technology, 44(8), pp.3169-3174.
